# Serum Liposoluble Vitamins (A, D, E) in Dogs with Chronic Biliary Tract Diseases Versus Healthy Dogs

**DOI:** 10.3390/vetsci12121195

**Published:** 2025-12-12

**Authors:** Verena Habermaass, Francesco Bartoli, Eleonora Gori, Aurora Cogozzo, Alessio Pierini, Paola Anna Erba, Chiara Mariti, Simonetta Citi, Caterina Puccinelli, Veronica Marchetti

**Affiliations:** 1 Department of Veterinary Sciences, Veterinary Teaching Hospital “Mario Modenato”, University of Pisa, Via Livornese Lato Monte, San Piero a Grado, 56122 Pisa, Italy; verena.habermaass@phd.unipi.it (V.H.); chiara.mariti@unipi.it (C.M.); simonetta.citi@unipi.it (S.C.); caterina.puccinelli@unipi.it (C.P.);; 2 Department of Translational Research and New Technologies in Medicine and Surgery, University of Pisa, Via Savi 10, 56126 Pisa, Italy

**Keywords:** fat-soluble vitamins, 25-hydroxyvitamin D, α-tocopherol, retinol, biliary disease, cholestasis

## Abstract

Humans with chronic biliary tract disease (CBTD) have low serum liposoluble vitamins (A, D, E). Few studies have been performed in veterinary medicine to evaluate whether vitamins vary in canine CBTD. This study aimed to compare liposoluble vitamin between CBTD and healthy dogs. A total of 84 client-owned dogs with CBTD and 50 healthy dogs were included. CBTD diagnosis was based on clinical, blood biochemistry and abdominal ultrasound. Dogs with CBTD were divided into subgroups according to their cholestasis ultrasound severity. To measure vitamin concentrations, leftover serum samples were used. The 25-hydroxyvitamin D, α-tocopherol, and retinol, respectively, vitamin D, E, and A metabolites, were measured with HPLC. Both, 25-hydroxyvitamin D and α-tocopherol were significatively lower in CBTD than in healthy dogs. In contrast, retinol was higher in CBTD dogs. In CBTD dogs, no significant differences in vitamin concentrations considering ultrasound severity were found. Presence of biliary disease in dogs results in lower blood vitamins D and E, and higher vitamin A concentration. Lower vitamins D and E concentration could reflect a possible lipid malabsorption. The higher concentration of vitamin A could be in line with recent human studies, where retinol increases as an expression of dysregulated homeostasis during chronic liver disease.

## Simple Summary

Chronic biliary tract disease (CBTD) in dogs can affect how the body processes and absorbs fat-soluble vitamins, such as vitamins A, D, and E. While this relationship is well established in humans, it has been little explored in veterinary medicine. This study compared the levels of these vitamins in dogs with CBTD and in healthy dogs. We included 84 dogs with CBTD and 50 healthy controls. The diagnosis of CBTD was based on clinical signs, blood tests, and abdominal ultrasound. Dogs with CBTD were further divided according to the severity of cholestasis observed on ultrasound. Vitamin levels were measured in leftover serum samples. Concentrations of 25-hydroxyvitamin D (vitamin D), α-tocopherol (vitamin E), and retinol (vitamin A) were determined using HPLC. Dogs with CBTD had significantly lower levels of vitamins D and E compared to healthy dogs, while vitamin A levels were higher. Within the CBTD group, vitamin levels did not differ according to disease severity. These findings suggest that chronic biliary disease in dogs is associated with reduced blood levels of vitamins D and E, possibly due to impaired fat absorption. The increase in vitamin A may reflect altered metabolic regulation, as observed in humans with chronic liver disease.

## 1. Introduction

Through bile production and secretion, the biliary system plays a key role in digestive processes and metabolic homeostasis. Cholestasis consists of an impaired intra- or extra-hepatic bile secretion, flow, or both, resulting in the reduction in bile flow into the small intestine [1,2]. Alteration in biliary secretion and bile acids metabolism characterize the cholestatic hepatopathy [2]. The absorption of liposoluble vitamins from the diet requires the detergent actions of bile acids contained in bile. Bile acids are amphipathic sterols synthesized from cholesterol in the liver and secreted into the intestine. Bile flow, and a sufficient concentration of bile acids in the intestinal lumen, are thus essential to emulsify lipids and for the uptake of lipids and fat-soluble compounds, which is why their metabolism may be impaired in cholestatic patients [3]. After intestinal absorption, liposoluble vitamins, through chylomicrons, are transported from the gut to the liver. Intestinal dysbiosis and inflammation, both in human and canine patients, are also known to play a key role in the digestion and absorption of nutrients [4,5]. The alteration of the enterohepatic circulation of bile acids, which are important lipid emulsifiers, could contribute to malabsorption [6]. Human patients with chronic cholestasis generally have lower serum liposoluble vitamins (A, D, E) if compared to healthy controls [7,8]; this has multiple clinical implications, such as neurological, vision, and skeletal disorders [9,10,11,12,13,14,15]. Chronic cholestasis increases the risk of fat-soluble vitamin deficiency, most commonly vitamin A and D, particularly in patients with serum bilirubin >2 mg/dL [8]. However, vitamin E is also reported to be low in patients with primary biliary cirrhosis [7,8,9,10,11,12,13,14,15]. The oral administration of fat-soluble vitamins has been shown to be effective and safe in pediatric patients with cholestasis [16,17,18].

A limited number of recent studies have investigated fat-soluble vitamin concentrations in dogs with various gastrointestinal disorders (Table 1). In dogs with exocrine pancreatic insufficiency, serum concentrations of vitamin A and E were consistently lower than in healthy controls [18]. Whereas vitamin D was mainly found to be low in dogs with chronic enteropathy and protein-losing enteropathy [19]. Conversely, vitamin A was found to be higher in dogs with chronic enteropathy while serum vitamin E levels do not seem to differ between healthy and affected dogs [20]. Few studies have investigated vitamin A, D, and E levels in dogs with chronic hepatobiliary disease. Vitamin D was reported to be lower in several conditions than in healthy control dogs, including the following: dogs with congenital biliary atresia, gallbladder mucocele, in a group of 16 dogs with different kinds of chronic liver diseases (including hepatic tumors like lymphoma, hepatocellular adenocarcinoma, miscellaneous hepatopathies, and cirrhosis), and in a group of 10 young dogs with congenital extrahepatic portosystemic shunt [21,22,23,24,25]. Vitamin A also tends to be low in dogs with chronic liver diseases and congenital extrahepatic portosystemic shunts [23,24,25].

Our hypothesis was that dogs diagnosed with chronic biliary tract diseases (CBTD) may present different concentrations of serum fat-soluble vitamins if compared to healthy dogs. In addition, we hypothesized that possible changes in fat-soluble vitamins may be related to ultrasonographic severity of biliary tract alteration. Thus, the aim of the present study was to assess the serum levels of fat-soluble vitamins A, D, and E in dogs with naturally acquired chronic biliary tract diseases and in healthy control dogs. We also investigated the vitamin levels in a group of CBTD dogs and the potential relationship with the severity of their ultrasonographic biliary cholestasis.

## 2. Materials and Methods

This was a retrospective case–control study on client-owned dogs with CBTD, of different breeds, sex, and weight, which were referred to the Internal Medicine Unit at the Veterinary Teaching Hospital of Pisa University, between 2021 and 2023; a group of healthy dogs was also included as a control. All methods were carried out in accordance with the ethical guidelines of the Department of Veterinary Sciences of Pisa and received favorable opinion by University of Pisa Ethics committee (No. 41/2020).

CBTD diagnosis was based on compatible medical history (icterus, hyporexia, or anorexia, vomiting, diarrhea lasting from more than four weeks), physical examination, hematology, blood biochemistry, and abdominal ultrasonography. To be included, dogs had to present concurrent clinical, biochemical, and ultrasonographic signs of biliary tract disease for more than 2 months. Specifically, two or more of the following laboratory alterations: alkaline phosphatase (ALP) >250 U/L (reference range 45–250 U/L), gamma-glutamyl transferase (GGT) >11 U/L (reference range 2–11 U/L), total bilirubin >0.3 mg/dL (reference range 0.07–0.3 mg/dL), cholesterol >280 mg/dL (reference range 120–280 mg/dL), and one or more concurrent ultrasonographic biliary tract alterations (such as biliary sludge, mobility of the biliary sludge, cholelithiasis, gallbladder wall thickness, intrahepatic biliary tree dilatation, mineralization of the intrahepatic biliary tree, and common biliary duct dilatation). Dogs with clinical and/or hematobiochemical evidence of liver failure (e.g., hyperammonemia, hypoproteinemia and hypoalbuminemia, hypocholesterolemia or hypoazotemia, or a combination of these) were excluded. Increased serum bile acids were not considered as exclusion criteria, since they can be considered secondary to biliary cholestasis, rather than an expression of reduced liver function [26]. Dogs with significative clinical, anamnestic, or biochemical comorbidities (renal, cardiac, oncologic, and primary chronic enteropathy) were excluded.

A group of healthy dogs were included as controls (C). In the control group (C group), healthy blood donors and healthy dogs owned by staff members of the Veterinary Teaching Hospital of Pisa were included. Blood donors were selected according to the Italian Ministry of Health Guidelines [27]. Each animal was classified as healthy after an evaluation of clinical history, physical examination, hematobiochemical profile, urinalysis (including protein/creatinine ratio), and fecal parasitological examination.

For both groups, dogs with a history of liposoluble vitamin supplementation in the last six months were excluded. According to the routinary requirements, the dogs were fasted for at least 12 h before blood sampling to avoid artefactual alterations in the concentrations of the analytes considered.

Abdominal ultrasound was performed by radiologists with a Canon Aplio a CUS-AA000 (Canon Medical Systems Europe B.V., Zoetermeer, The Netherlands), with a 7.5 MHz microconvex probe and a 12 MHz linear probe. All dogs underwent fasting at least 12 h before the procedure. During the ultrasound, all lobes of the liver and the biliary system were assessed. DICOM images and video files were stored in the Veterinary Teaching Hospital database and retrospectively reviewed by one author (S.C.). One individual veterinarian (S.C.) retrospectively assessed the images and videos to prevent an impact of interobserver variation on data. Ultrasonographic features were categorized according to abnormalities of the gallbladder (GB) wall and/or content, of the intrahepatic biliary tree, and of the common bile duct [28,29,30,31]. A previously used semi-objective scoring system was used [32]. The ultrasound parameters and their assigned scoring system are reported in Table 2. The grade of biliary sludge (BS) was defined as 1 for BS occupying <25% of the GB area, 2 for 26% to 50% of the GB area, 3 for 51% to 75% of the GB area, 4 for >75% of the GB area, and 5 for gallbladder mucocele [29,30,31]. Based on the presence of these alterations, the dogs were classified with mild (1–2), moderate (3–4), and severe (>5) biliary tract abnormalities. All dogs included in the study had to have at least one alteration. CBTD group was subsequently divided in subgroups according to ultrasonographic features described above.

Serum concentrations of 25-hydroxyvitamin D (25(OH)D), α-tocopherol, and retinol—corresponding to vitamins D, E, and A, respectively—were quantified using high-performance liquid chromatography (HPLC, JASCO Europe, Cremella, Italy). All samples were processed and analyzed under standardized and identical conditions to ensure uniformity across the study groups. After collection, blood samples were centrifuged within 30 min. Serum aliquots (0.5 mL) were stored at −18 °C within 24 h and transferred to −80 °C within 5 days, maintaining uninterrupted cold chain conditions. All dogs were fasted for at least 12 h prior to sampling. No serum sample used in the analysis was stored for more than 24 months [33]. For HPLC analysis, 200 µL of thawed serum was mixed with 200 µL of absolute ethanol and vortexed for 10 s. Lipophilic compounds were extracted twice with 1.5 mL of HPLC-grade hexane. The mixture was centrifuged at 15,000 × *g* for 5 min at room temperature. The organic phase was collected, evaporated under nitrogen in the dark to prevent photodegradation, and reconstituted in 200 µL of methanol. A volume of 100 µL was injected for chromatographic analysis. Chromatographic separation was performed on a Luna LC18 column (150 × 2.6 mm I.D., 5 µm particle size; Phenomenex Inc., Torrance, CA, USA) maintained at 25 °C. The mobile phase consisted of 95% methanol in water, delivered isocratically at a flow rate of 0.6 mL/min. Detection wavelengths were set at 280 nm for 25(OH)D, 292 nm for α-tocopherol, and 325 nm for retinol. Identification of each analyte was based on retention time compared to analytical standards, prepared in methanol, and run under the same conditions. Calibration curves were prepared in methanol, the same solvent used for the samples using increasing concentrations (1, 5, 10, 15, 25, 50, 100, and 150 ng/mL) for each analyte. Each point was analyzed in duplicate. Detector response showed excellent linearity, with coefficients of determination (R^2^) exceeding 0.98 for all analytes. The method demonstrated adequate sensitivity for the detection and quantification of all analytes within the physiologically relevant concentration ranges observed in canine serum. The linear range for calibration curves spanned from 1 to 150 ng/mL for all analytes, covering the expected biological concentrations. The lowest calibration point (1 ng/mL) was consistently detectable with a signal-to-noise ratio >10:1 and was used as the practical limit of quantification for this study. All measured sample concentrations fell well above this threshold, ensuring reliable quantification. Intra-assay precision, determined by analyzing multiple replicates of quality control samples, showed coefficients of variation below 5% for all analytes. Inter-assay precision, evaluated over multiple analytical runs, showed coefficients of variation below 8% for all analytes. The recovery rates were determined by spiking known concentrations of each analyte into pooled plasma from a healthy donor dog. The spiked samples underwent the full extraction protocol described above. The calculated average recovery rates were 94.5% for 25(OH)D (CV 0.8%), 96.5% for α-tocopherol (CV 2.3%), and 96.8% for retinol (CV 1.3%). Importantly, these recovery rates were calculated for methodological validation only and were not used to adjust the measured concentrations, since the study design involved relative comparisons between groups. All serum samples were handled, stored, extracted, and analyzed under strictly identical conditions, ensuring internal methodological consistency and valid group-to-group comparisons. All serum specimens underwent the identical, internally validated HPLC-DAD workflow—encompassing uniform protein precipitation, centrifugation parameters, and elution volumes—and demonstrated robust intra- and inter-assay precision (%CV < 10%). Consequently, absolute recovery adjustments were deemed unnecessary for the relative group comparisons central to this study. Moreover, due to the use of a diode array detector (DAD, JASCO Europe, Cremella, Italy), without isotopically labeled internal standards—precluding the rigorous calibration required for true quantification—the assay is considered semiquantitative and results are interpreted solely as relative differences between experimental cohorts.

Statistical analysis was performed with GraphPad Prism, version 9.0 (GraphPad Software, San Diego, CA, USA). No a priori sample size calculation was performed. Normal distribution was investigated through the Kolmogorov–Smirnov test. Since non-normally distributed, data were expressed as median and range. The Mann–Whitney U-test was used to compare vitamin A, D, and E levels between healthy and CBTD groups. The Kruskal–Wallis test was used to evaluate differences in serum vitamins among biliary tract ultrasonographic alterations in CBTD. Results with *p* value <0.05 were considered statistically significant.

## 3. Results

### 3.1. Animals

Eighty-four client-owned dogs with CBTD were enrolled in the study. The majority of CBTD dogs were mixed-breeds (*n* = 28; 33%), followed by Dachshund (*n* = 6; 7%), Jack Russel Terrier (*n* = 5; 6%), Cavalier King Charles Spaniel (*n* = 4; 5%), Golden Retriever (*n* = 3; 3%), Shih Tzu (*n* = 3; 3%), Boxer (*n* = 2; 3%), Springer Spaniel (*n* = 2; 3%), Border Collie (*n* = 2; 3%), Bernese Mountain Dog (*n* = 2; 3%), Maltese (*n* = 2; 3%), German Shepherd (*n* = 2; 3%), Zwergpinscher (*n* = 2; 3%), West Highland White Terrier (*n* = 2; 3%), Cocker Spaniel (*n* = 2; 3%), and one of each (1%) of the following breeds: German Shorthaired Pointer, Basset Hound, Boston Terrier, Toy Poodle, Bullterrier, Flat Coated Retriever, Australian Shepherd, Papillon, Chihuahua, Fox Terrier, Lagotto Romagnolo, Maremma Sheepdog, Spinone Italiano, Weimaraner, Yorkshire Terrier, Pomeranian Spitz, and English Setter. Median age was 9 years (range 0.3–18), and sex was equally distributed with 43 female (29/43 neutered) and 41 male (8/41 neutered) dogs. Serum biochemical findings of the CBTD group are reported in Table 3.

Fifty healthy dogs were included in C group. The majority were mixed-breeds (*n* = 16; 32%), followed by Labrador Retriever (*n* = 5; 10%), French Bulldog (*n* = 3; 6%), Galgo (*n* = 2; 4%), Golden Retriever (*n* = 2; 4%), Jack Russel Terrier (*n* = 2; 4%), and one of each (2%) of the following breeds: Breton, American Staffordshire Terrier, Basset Hound, Dachshund, Boxer, Cavalier King Charles Spaniel, Bracco Italiano, Cocker Spaniel, Dobermann Pincher, Greyhound, Maremma Sheepdog, Australian Shepherd, German Shepherd, Zwergpinscher, Pitbull Terrier, Rottweiler, Shiba Inu, and West Highland White Terrier and Newfoundland. Median age was 3.8 years (range 0.3–13). In group C, 29 dogs were female (12/29 neutered) and 21 were male (6/21 neutered). CBTD dogs had a significantly higher median age than the healthy controls (*p* < 0.0001).

### 3.2. Vitamin A, D, and E Analysis

**Figure 1:**
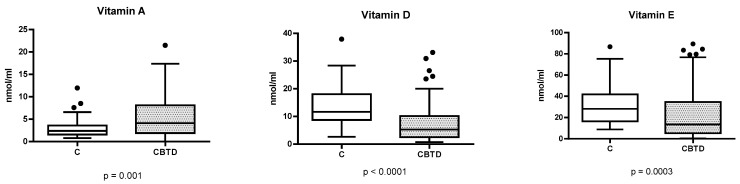
Differences in serum liposoluble vitamins D, E, and A (ng/mL) between dogs with chronic biliary tract disease (CBTD; *n* = 84) and healthy controls (C; *n* = 50). Box and Whiskers graph; black dots represent the outliers. Applied statistical tests: Mann–Whitney U test.

Considering fat-soluble vitamins, vitamins D and E were significatively lower in the group of CBTD than in group C: 6.84 ng/mL (0.76–33.12) vs. 11.68 ng/mL (2.65–37.93), *p* ≤ 0.0001, for 25-hydroxyvitamin D and 14 ng/mL (0.27–89.41) vs. 28.22 ng/mL (8.82–86.68) for α-tocopherol, respectively. Conversely, vitamin A was significantly higher in the CBTD group than in group C, specifically 4.05 ng/mL (0.02–21.48) vs. 2.37 ng/mL (0.87–11.97) (Figure 1).

**Figure 2:**
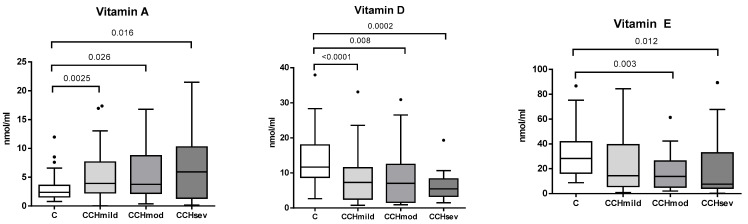
Differences in serum liposoluble vitamins D, E, and A (ng/mL) between dogs with chronic biliary tract disease (CBTD) according to ultrasonographic biliary stasis classification (mild, *n* = 51; moderate, *n* = 19 and severe, *n* = 14) and healthy controls (C, *n* = 50). Box and Whiskers graph; black dots represent the outliers. Applied statistical tests: Mann–Whitney U test. Significant differences are reported within the graph.

Based on ultrasonographic alterations, 51 dogs out of the 84 CBTD (61%) dogs presented mild ultrasonographic signs of biliary tract abnormality, whereas 19 dogs (22%) presented moderate signs, and 14 dogs (17%) had ultrasonographic features suggestive of severe biliary tract disease. No significant difference in vitamin levels was found in dogs with different CBTD severities as assessed by the ultrasonographic biliary stasis classification (mild, moderate, and severe). Specifically, the median vitamin D levels were 7.28 ng/mL (range: 0.76–33.12) in dogs with mild CBTD, 7.06 ng/mL (0.92–30.91) in moderate cases, and 5.46 ng/mL (1.5–19.34) in severe cases (*p* = 0.91). For vitamin E, levels were 14.36 ng/mL (0.79–84.49) in mild, 13.83 ng/mL (2.07–61.35) in moderate, and 7.6 ng/mL (0.27–89.41) in severe CBTD (*p* = 0.63). Regarding vitamin A, serum concentrations were 3.93 ng/mL (0.02–17.36), 3.76 ng/mL (0.36–16.78), and 5.91 ng/mL (0.15–21.48) in mild, moderate, and severe cases, respectively, (*p* = 0.88). Figure 2 shows the results of serum liposoluble vitamins D, E, and A (ng/mL) in healthy controls and mild, moderate, and severe ultrasonographic CBTD.

## 4. Discussion

As expected, vitamins D and E were lower in the CBTD group than in healthy dogs, while vitamin A was higher. In addition, the concentrations of serum fat-soluble vitamins A, D, and E did not differ significantly among CBTD subgroups with mild, moderate, or severe cholestasis.

Given its long half-life (2–3 weeks), 25(OH)D is considered the most accurate measurement to assess vitamin D status in both human and veterinary medicine [34,35]. In human patients with liver diseases, many mechanisms can decrease vitamin D levels. Decreased cutaneous vitamin D synthesis [36], common in humans, cannot be considered in dogs as this species cannot synthesize vitamin D at the skin level because of the lack of free 7-dehydrocholesterol in canine epidermal cells [37,38]. A reduced hepatic 25-hydroxylation [39] and decreased hepatic synthesis of vitamin D-binding protein [40] due to reduced liver function, as well as malabsorption due to prolonged cholestasis [7,8], are considered the main causes of this deficiency. The liver is critical for normal vitamin D levels, being involved in the conversion into its active forms [40] and, through bile, in the digestion and absorption of fats and fat-soluble vitamins, including vitamin D. During cholestasis, the reduced flow of bile leads to less vitamin D absorbed from the diet [7,8,41]. This process is likely to be particularly relevant in dogs, who rely on diet for the provision of vitamin D.

Although previous studies have already highlighted the relevant role of liver diseases in reducing serum levels of vitamin D (e.g., in cats with hepatic lipidosis [20] and in dogs diagnosed with different chronic liver diseases including neoplasia and cirrhosis [23]), most studies focused on hepatocellular modification. Meanwhile, there is a general lack of information regarding the alteration in liposoluble vitamins in biliary disease. In a case report of a young dog with congenital biliary atresia, reduced absorption is suggested as the possible cause of vitamin D deficiency and rickets [22]. A recent study, which considered cholestasis as a possible cause of decreased vitamin D, showed significantly lower serum vitamin D concentrations in 62 dogs with gallbladder mucocele compared to healthy controls. Moreover, vitamin D level showed a positive correlation with the developmental stage of gallbladder mucocele determined by ultrasonography [24]. In the current study, a negative non-significant trend was found consisting in dogs with severe ultrasonographic biliary cholestasis showing lower vitamin D levels than dogs with mild and moderate signs of biliary stasis. Despite the lack of statistical significance, further studies should investigate the levels of vitamin D in dogs with severe biliary cholestasis. The low level of vitamin D in cholestatic dogs may result from malabsorption due to chronic biliary stasis; however, it may also result from decreased binding capacity due to impaired synthesis of transport proteins or a compromised 25-hydroxylation of vitamin D due to chronic liver disease [21]. Nevertheless, an experimental study conducted on dogs with common bile duct ligation suggests that vitamin D deficiency in cholestatic liver disease is most probably due to malabsorption rather than reduction in hepatic 25-hydroxylation [21]. The current study seems to confirm that malabsorption is the most likely cause of vitamin D reduction, as dogs with evidence of liver failure, such as dogs with a protidic deficit evident from panhypoproteinemia, were excluded. The oral administration of vitamin D has proven effective and safe in human patients with cholestasis [16,17]. In contrast, few studies have evaluated serum vitamin D concentrations in dogs with CBTD or examined the potential benefits of calcitriol supplementation in affected dogs, despite the growing interest in the role of vitamin D beyond skeletal health [42]. Vitamin D supplementation has been explored in dogs under various conditions. In recent intervention studies involving healthy animals, cholecalciferol (vitamin D3) has been the most frequently administered form. These trials indicate that daily oral D3—such as 50 IU/kg/day for six weeks—consistently elevates serum 25-hydroxyvitamin D [25(OH)D] levels without causing short-term biochemical or clinical adverse effects [43].

Vitamin E includes a group of fat-soluble compounds, such as tocopherols (alpha-tocopherol, beta-tocopherol, gamma-tocopherol, and delta-tocopherol) and tocotrienols (alpha-tocotrienol, beta-tocotrienol, gamma-tocotrienol, and delta-tocotrienol), with alpha-tocopherol showing the highest biological activity. Vitamin E supplementation in canine chronic liver disease is recommended due to its antioxidant properties [44,45], despite the limited information on its effectiveness [46]. This recommendation is mostly based on its effectiveness in human patients with NALFD (Nonalcoholic Fatty Liver Disease) [47,48] and in the prevention and treatment of infants with chronic cholestasis due to biliary atresia [48,49].

In contrast to a previous study [23], we observed a reduction in vitamin E serum levels in CBTD dogs compared to healthy dogs. This disagreement could be due to differences in the characteristics of the study population, since we included only dogs with biliary chronic cholestasis while in this other study from, 2013 [23], they studied 16 dogs with different kinds of chronic liver diseases. This suggests that malabsorption due to alteration of the biliary physiology and chronic cholestasis could play a significant role in serum vitamin E deficiency during canine chronic liver disease. In addition, we found that vitamin E did not differ significantly among dogs with mild, moderate, and severe ultrasonographic signs of cholestasis. This could also be due to the interindividual variability of vitamin E in relation to its complex metabolism in the oxidative processes, including chronic liver disease. However, since oxidative stress is recognized as a pathological mechanism in canine chronic liver disease [45] and cholestasis-related vitamin E deficiency was detected in the present study, the dietary supplementation of vitamin E could be considered as a therapeutic approach, and further studies on its usefulness are necessary. Other studies in veterinary medicine have assessed the efficacy and safety of vitamin E supplementation—using various dosing protocols—in dogs affected by different conditions, including dermatologic and reproductive disorders. In these studies, vitamin E was demonstrated to reduce oxidative stress and provide clinical benefits [50,51]. Vitamin A includes different kinds of retinoids and their precursor forms. Mammals are not able to completely synthesize vitamin A, thus they depend on the nutritional uptake of vitamin A or β-carotenes, which are prerequisites for vitamin A synthesis [52]. Vitamin A is mainly stored in the liver as retinyl palmitate esters, precisely in hepatic Ito cells (or hepatic stellate cells). During liver injury, these cells lose vitamin A and contribute to hepatic fibrosis [53,54,55,56]. Vitamin A deficiencies in human chronic cholestatic diseases have been frequently reported [7,57], and an altered retinol metabolism appears to be involved in the complex process of hepatic fibrosis [58]. In rat models with cholestasis and liver fibrosis, vitamin A supplementation in vivo seems to attenuate hepatic fibrosis [59]. However, especially in cholestatic diseases, serum retinoid levels seem to differ significantly from hepatic levels. If low serum retinoid levels were only caused by the reduced absorption, hepatic retinoid levels would be expected to be low to normal in human patients with primary biliary cirrhosis. However, hepatic vitamin A accumulation has been found in hepatic stellate cells with an impaired release from the liver [60], which has also been found in canine patients with chronic liver disease [23].

In contrast to the common findings in human cholestatic patients and to preliminary evidence in dogs with histological chronic liver disease [23], in the present study vitamin A was found to be higher in CBTD dogs compared to healthy controls. Moreover, although not statistically significant, a trend was found for vitamin A to be higher in dogs with severe and moderate ultrasonographic biliary tract abnormalities compared to dogs with mild signs. Interestingly, this result is consistent with the evidence of increased retinol found in dogs with chronic enteropathy. As expressed by the authors, the serum retinol concentration may not reflect the body storage levels of vitamin A [20].

To the best of our knowledge, this is the first report of increased serum retinol in canine patients with chronic cholestatic disease. Indeed, this unexpected result is in line with recent evidence in human patients. In fact, it has been reported that aldo-keto reductase family 1 member B10 (AKR1B10), which is a key enzyme of the retinol metabolism [60,61,62], is overexpressed in the liver of patients with nonalcoholic steatohepatitis (NASH) compared to those with simple hepatic steatosis (SS) and controls [63,64]. It has also been demonstrated that patients with NAFLD [65], NASH, and SS showed higher serum retinol levels compared to liver donors [66], which is due to the different hepatic expression pattern of genes related to retinol metabolism. The highest degree of overexpression was observed for AKR1B10 in NASH versus both SS and liver donors, but also the enzymes ALDH1A2 and ALDH1A3 (Aldehyde Dehydrogenase 1 Family Member A2 and A3), which are responsible for retinoic acid synthesis from retinaldehyde, were inversely correlated with plasma retinol levels. The upregulation of these genes could be due to the oxidative stress related to the liver disease [67,68,69]. Although findings on serum levels of vitamin A levels in NAFLD are contrasting, there is some evidence that retinol may be higher if liver disease is present [70]. We hypothesize that a similar mechanism could be present in dogs and explain our results. However, further studies are needed to better understand the metabolic pattern of vitamin A in canine chronic hepatobiliary disease.

The results of this study should be considered in the light of some possible biases. Vitamins have a complex metabolism, and it may be difficult to discern whether other comorbidities, also related to the liver disease itself, may have affected the vitamin serum levels (e.g., secondary chronic enteropathy, inflammatory processes, endocrinopathies [71,72]). Moreover, in dogs, primary biliary pathologies are rare, contrary to human Primary Biliary Cholangitis and Primary Sclerosing Cholangitis, and information on the effect of some common comorbidities (e.g., endocrinopathies) on serum vitamin levels is lacking. Similarly, no age or breed-matching was performed, and this represents a potential confounding factor, even in the absence of the solid literature reporting differences in serum fat-soluble vitamins in dogs according to age or breed. To fill this current bibliographic gap, further studies are necessary.

In addition, the reduced production of proteins and specific binding proteins during liver disease could also lower the circulating levels of vitamins, making it difficult to evaluate the effective role of biliary stasis. The interpretation of serum vitamin concentrations has some limitations, since serum levels do not always reflect the vitamin levels in storage tissues. Vitamin serum levels are also influenced by diet and feed intake [73,74,75]. Although we excluded dogs receiving non-dietary vitamin supplementations of fat-soluble vitamins, the diet in the present study was not standardized either in the CBTD group or control dogs. For future studies, it would be advisable to standardize or record the vitamin content of the diet. Moreover, even in absence of current solid scientific evidence, we cannot exclude that other ongoing treatments in our population may have affected the concentration of serum vitamins. Although the used ultrasound scoring system has been previously published, formal intra-observer and inter-observer reliability assessments were not reported in that study. Consequently, such validation was not available for this study either. Another limit may be represented by the absence of abdominal ultrasound examination of healthy controls, thus abnormal findings in the biliary tract (e.g., biliary sludge) cannot be conclusively ruled out. Nevertheless, having significant CBTD in the absence of clinical signs and unremarkable biochemical evaluation is unlikely. The lack of hepatic histological assessment for all the dogs is a limitation, since it could more precisely describe the extension, type, and severity of cholestasis and thus, be potentially related to the modification in the liposoluble vitamins concentration. In conclusion, this observational work should serve as a foundation for future targeted studies, including vitamin K evaluation, aimed at clarifying the underlying physiopathologic and biochemical mechanisms, as well as for subsequent clinical trials designed to assess the actual safety and potential benefits—or the need for restriction—of specific vitamin supplementations in dogs with chronic biliary disease.

## 5. Conclusions

As it happens in human patients, in our study vitamins D and E were lower if the dog presented a chronic biliary tract disease, suggesting a possible lipid malabsorption. Further studies evaluating safety and potential benefits of vitamins D and E supplementation in canine patients with chronic biliary tract disease are necessary.

In contrast, vitamin A was higher in dogs with chronic biliary tract disease. This result is in line with recent human studies, where retinol was found to increase as an expression of dysregulated vitamin A and related metabolites homeostasis in the Ito cells and hepatocytes. Further studies are needed in dogs with liver disease to better understand the relevance of vitamin A dysmetabolism and its underlying mechanism.

## Figures and Tables

**Table 1:** Serum levels of Vitamin A, D, E found in dogs with various gastrointestinal diseases reported in the veterinary literature.

Vitamin	Serum Levels Compared to Healthy Dogs
Vitamin A	↑ Chronic Enteropathy [20] ↓ Miscellaneous Chronic Liver Diseases [23] ↓ Congenital Extrahepatic Portosystemic Shunt [25] ↓ Exocrine Pancreatic Insufficiency [18]
Vitamin D	↓ Chronic Enteropathy (Protein Losing Enteropathy) [19,20] ↓ Miscellaneous Chronic Liver Diseases [23] ↓ Congenital Biliary Atresia, Gallbladder Mucocele [22,24] ↓ Congenital Extrahepatic Portosystemic Shunt [25] ≅ Exocrine Pancreatic Insufficiency [18]
Vitamin E	≅ Chronic Enteropathy [20] ≅ Miscellaneous Chronic Liver Diseases [23] ↓ Exocrine Pancreatic Insufficiency [18]

Legends: ↓: reduced; ↑: increased; ≅: approximately equal to.

**Table 2:** Ultrasonographic biliary abnormalities evaluated in CBTD dogs enrolled in this study.

Score Type	Ultrasound Abnormalities	Points					
		0	1	2	3	4	5
Gallbladder	Biliary sludge	Absent	Grade 1	Grade 2	Grade 3	Grade 4	Mucocele
	Mobility of the biliary sludge	Gravity dependent	\	Non-gravity dependent	\	\	\
	Cholelithiasis	No	\	Yes	\	\	\
	Gallbladder wall thickness	<2 mm	>2 mm	Cysts/mineralitazions/polypoid lesions	\	\	\
Intrahepatic biliary tree and common bile duct	Intrahepatic biliary tree dilatation	No	\	Yes	\	\	\
	Mineralization of the intrahepatic biliary tree	No	Yes	\	\	\	\
	Common bile duct dilatation	No	Yes	\	\	\	\

**Table 3:** Serum biochemistry in healthy C dogs (*n* = 50) and CBTD dogs (*n* =84).

Parameter	C Dogs	Median (Range)	Reference Interval
ALP	156 (53–243)	768 (31–45000)	45–250 (U/L)
GGT	3.2 (0.2–6.8)	15.5 (0.1–751.6)	2–11 (U/L)
AST	21 (17.2–38)	73.5 (12–1137)	15–40 (U/L)
ALT	64 (41–67.3)	164 (4.9–4773)	40–70 (U/L)
Total bilirubin	0.12 (0.03–0.25)	0.27 (0.09–30)	0.07–0.3 (mg/dL)
Cholesterol	195 (132–269)	283 (37–816)	120–280 (mg/dL)
Total serum proteins	6.5 (5.8–7.6)	6.3 (4.3–9.3)	5.8–7.8 (g/dL)
Albumin	3.4 (2.8–4.1)	3.3 (2–4.8)	2.6–4.1 (g/dL)

## Data Availability

The original contributions presented in this study are included in the article. Further inquiries can be directed to the corresponding author.

## Abbreviations

The following abbreviations are used in this manuscript:

CBTD	Chronic biliary tract disease
C	Controls
25(OH)D	25-hydroxycholecalciferol
GB	Gallbladder
BS	Biliary sludge
ALP	Alkaline phosphatase
GGT	Gamma-glutamyl transferase
AST	Aspartate aminotransferase
ALT	Alanine aminotransferase
SS	Simple steatosis
NAFLD	Non-alcoholic fatty liver disease
NASH	Non-alcoholic steatohepatitis
AKR1B10	aldo-keto reductase family 1 member B10
ALDH1A2	Aldehyde Dehydrogenase 1 Family Member A2
ALDH1A3	Aldehyde Dehydrogenase 1 Family Member A3
